# Prevalence of intestinal parasites and associated factors among patients with HIV/AIDS at the anti-retroviral treatment clinic of Mizan-Tepi University Teaching Hospital, Southwest Ethiopia

**DOI:** 10.3389/fpubh.2024.1451757

**Published:** 2024-11-27

**Authors:** Mengistu Abayneh, Yosef Habtemariam, Tadesse Duguma, Mitiku Abera

**Affiliations:** ^1^Department of Medical Laboratory Sciences, College of Medicine and Health Science, Mizan-Tepi University, Mizan Teferi, Ethiopia; ^2^Department of Medicine, College of Medicine and Health Science, Mizan-Tepi University, Mizan Teferi, Ethiopia

**Keywords:** prevalence, intestinal parasite, people with HIV/AIDS, Ethiopia, ART clinic

## Abstract

**Background:**

Intestinal parasitic infections remain very common, particularly in areas with a high prevalence of immune-compromised patients, such as HIV/AIDS patients. The purpose of this study was to determine the prevalence of intestinal parasites and associated factors in people living with HIV/AIDS at an ART clinic in Mizan-Tepi University Teaching Hospital, southwest Ethiopia.

**Method:**

A cross-sectional survey was conducted from July to September 2021. A total of 191 adult people living with HIV/AIDS participated in this study. Data on socio-demographic, clinical, and other risk factors were collected using a structured questionnaire. Stool samples were collected and processed using a direct wet mount, formol-ether concentration, and modified Ziehl-Nelson staining techniques. The data were analyzed using the Statistical Package for Social Sciences Version 25 software.

**Results:**

Among 67 adult individuals living with HIV/AIDS, the prevalence of intestinal parasites was 35.1%. Specifically, 31.5% (45/143) of patients on antiretroviral therapy (ART) and 45.8% (22/48) of ART-naïve patients were infected. The distribution of intestinal parasites was as follows: protozoa were found in 14.7% of ART-treated patients and 22.9% of ART-naïve patients; helminths in 15.4% of ART-treated patients and 16.7% of ART-naïve patients; and opportunistic parasites in 1.4% of ART-treated patients and 6.25% of ART-naïve patients. Significant associations with a higher prevalence of intestinal parasites were observed for a CD4 count <200 cells/mm^3^ (Adjusted Odds Ratio [AOR] = 3.77; 95% Confidence Interval [CI]: 1.01–13.15; *p* = 0.04), consumption of unwashed raw vegetables (AOR = 3.29; 95% CI: 1.23–8.86; *p* = 0.02), and residing in rural areas (AOR = 2.34; 95% CI: 1.27–4.32; *p* = 0.01).

**Conclusion:**

The findings indicate that a significant proportion of adults living with HIV/AIDS are affected by intestinal parasites, with a notably higher prevalence among ART-naïve patients compared to those on ART. Factors such as a low CD4 count, consumption of unwashed raw vegetables, and rural residence are associated with increased risk of intestinal parasite infections. These results underscore the importance of improving hygiene practices and access to healthcare, particularly in rural areas, to reduce the burden of parasitic infections among individuals living with HIV/AIDS.

## Introduction

Parasitic infections, particularly intestinal parasites, are among the most widespread human infections worldwide. They are the main problem in developing countries, as they have been noticeably heightened with the coexistence of a large burden of Human Immunodeficiency Virus/ Acquired Immunodeficiency Syndrome (HIV/AIDS) and malnutrition in the area. Globally, approximately 38 million people were living with HIV in 2020, with sub-Saharan Africa accounting for nearly 67% of these cases. In Ethiopia, about 620,000 people are estimated to be living with HIV/AIDS, making it one of the countries with a significant burden of the disease. The coexistence of HIV/AIDS with parasitic infections and malnutrition further exacerbates health outcomes in these regions. For instance, it is estimated that more than 60% of the world’s population is infected with intestinal parasites, which significantly contribute to morbidity due to intestinal infections (1–4).

The gastrointestinal problem resulting from opportunistic parasitic infection in people living with HIV/AIDS has dramatically decreased in countries where antiretroviral agents are widely available. However, in most African countries where patients have low access to Antiretroviral Therapy (ART), intestinal pathogens still represent a frequent cause of diarrhea, wasting, and weight loss. Evidence shows that Human Immunodeficiency Virus (HIV)-infected patients are the most vulnerable risk group for acquiring parasitic infections, and about 85% of acquired immunodeficiency syndrome (AIDS) patients die as a result of AIDS-related infections rather than the HIV infection itself. These opportunistic infections most commonly occur in the later stages of HIV infection, when the number of Cluster of Differentiation 4 T cells (CD4 T cells) has declined mostly below 200/mm^3^ (5–8).

Intestinal parasitic infections are transmitted to humans through soil contaminated by human feces, mostly in areas where sanitation is poor. The infection leads to anemia, vitamin A deficiency, stunted growth, malnutrition, intestinal obstruction, and impaired development. The most commonly reported intestinal parasites are *Ascaris lumbricoides, hookworms, Trichuris trichiuria, Giardia lamblia, Entamoeba histolytica*, and *Schistosoma* species are the most common intestinal parasites. Among those opportunistic pathogens, *Isospora belli, Cryptosporidium parvum, Cyclospora cayetanenis*, and *Microsporidia* species are increasingly reported as causes of enteritis and as opportunistic pathogens in immune-compromised individuals (9–12).

Despite ART improving the quality of life and reducing the occurrence of opportunistic infections due to immunosuppression, malnutrition, poor waste management, ignorance (illiteracy), unhygienic sources of drinking water, and depleted CD4 counts among people living with HIV/AIDS are the most common determinants of parasitic infection (8, 11, 12). As Ethiopia is one of the developing countries in the world, it is categorized under countries with a high prevalence of parasitic infections. For instance, according to the studies, the average prevalence of parasitic infections was 39.6% (13–16), and the estimated pooled prevalence in HIV/AIDS patients was 39.2% (17). The severity and magnitude of IP in people living with HIV require attention and study, especially in countries like Ethiopia where there is high HIV/AIDS and parasite prevalence. For this reason, this study was conducted to determine the magnitude of intestinal parasites and risk factors among people living with HIV/AIDS at the ART clinic of Mizan-Tepi University (MTU) Teaching Hospital, southwestern Ethiopia. The finding will help to sensitize local and national responsible bodies as a prerequisite for updating strategies in the prevention and control of intestinal parasitic infection among people living with HIV/AIDS.

## Methodology

### Study area and period

This study was conducted at MTU teaching hospital from July to September 30, 2021. In the southwest direction, the hospital is located 591 km from Addis Ababa, the capital city of Ethiopia. It is the second teaching hospital with more than 139 beds in the southwestern part of the country, which provides services to approximately 5 million people from four catchment zones such as BENCH SHEKO, KEFA, SHEKA, and MAJANG as referral centers. Up to 350 clients visit the hospital daily for different services and more than 750 HIV-positive patients were taking ART services.

### Study design and subjects

A cross-sectional descriptive study design was conducted to determine the prevalence of intestinal parasites among HIV-positive patients who were attending the ART clinic at the MTU teaching hospital in southwest Ethiopia.

### Inclusion and exclusion criteria

#### Inclusion criteria

Adults aged 18 years and older.

Individuals diagnosed with HIV/AIDS.

Patients attending the ART clinic at MTU Teaching Hospital during the study period.

Individuals who provided informed consent to participate in the study.

#### Exclusion criteria

Individuals who were unwilling or unable to provide informed consent.

Patients who were unable or unwilling to provide stool samples.

### Operational definitions

#### Prevalence of intestinal parasites

The percentage of patients with HIV/AIDS irrespective of their ART status at the ART clinic of MTU University Teaching Hospital who test positive for one or more intestinal parasites during a specified study period.

#### ART patients

Individuals who have been diagnosed with HIV and have started ART. This includes anyone currently undergoing ART treatment.

#### ART naïve patients

Individuals who have been newly diagnosed with HIV and have not yet started ART. These are typically people screened at voluntary counseling and testing (VCT) centers who are ready to begin ART.

#### People living with HIV/AIDS

This term encompasses all individuals who have been diagnosed with HIV, regardless of whether they have started ART or are newly diagnosed and ready to start ART.

### Sample size and sampling technique

The required sample size was calculated using a single population proportion formula by assuming that confidence interval of 95%, a margin error of 5%, z statistic for confidence level (z = 1.96 at 95% CI), and the prevalence of intestinal parasites among people with HIV (p) as 13.9% (18), which is reported in previous study in Ethiopia, *n* = sample size was calculated as the followig.


$$\mathrm{Therefore}:n=\frac{{\left(Z\alpha /2\right)}^{2}p\left(1-p\right)}{d^{2}}$$

$$\mathrm{Therefore}:n=\frac{{\left(1.96\right)}^{2}\times 0.139\left(1-0.139\right)}{{\left(0.05\right)}^{2}}=184$$

By assuming a 5% non-respondent rate, the final sample size for this study was 191.

### Data collection

#### Socio-demographic and clinical data collection

During the implementation of the data collection, the study subjects were asked about their willingness to participate in the study. Two trained BSc nurses who were working at the ART clinic were recruited for the data collection. A structured questionnaire (face to face interview) was administered to collect socio-demographic (age, sex, residence, marital status, and educational status) and other risk factors data, such as hand washing practice, consuming raw food, owning domestic animals, the availability and usage of latrines, shoe wearing habits, and the source and treatment of water for drinking. Clinical information on the level of CD4+ T cell count, viral load, and ART status was obtained from patients’ medical records.

### Laboratory sample collection and processing

A laboratory technologist collected a single fresh stool sample from people living with HIV/AIDS attending at ART clinic of MTU Teaching Hospital. All the patients were oriented and provided with appropriate leak-proof stool cups to bring a 4-5 g of stool specimens. Each sample was labeled with a specific code number and taken to the parasitology laboratory of the MTU teaching hospital for parasitological analysis.

### Sample processing

The stools were examined both macroscopically and microscopically. Macroscopically, the stool samples were inspected for appearance; while microscopically they were examined using direct iodine mount smear preparation, formol-ether concentration, and the modified Ziehl-Neelson technique (19–21). During the formol-ether concentration technique, 1 g of stool was mixed with 7 mL of formalin-saline in a clear conical centrifuge tube. After collecting the sieved suspension in another conical test tube, 3 mL of diethyl ether was added, mixed for 1 min, and then centrifuged for 2 min at approximately 3,000 rpm. The supernatant (i.e., the ether, fecal debris, and formol water) was discarded, and the remaining small sediment was mixed well by shaking. About a drop of the sediments was transferred to a slide, to which a drop of iodine solution was added and covered with a cover slide. Finally, the preparations were examined under the light microscope with the 10X and 40X objectives (19–21). A modified Ziehl-Neelsen staining technique was used to detect *Cryptosporidium parvum* and *Isospora belli*. Hence, thin smears were prepared directly from sediment obtained by the formol ether concentration technique and then allowed to air dry and fixed with methanol for 3 min. Then the smears were flooded with carbon fuchsine for 15 min. The slide was washed with tap water and discolored with 1% acid-alcohol for 15 s. The slide was then rinsed in tap water and flooded with 0.5% methylene blue for 30 s. Wash off the slides with water, allow the smear to dry, and observe under a light microscope with 100X objectives (20, 21).

### Data analysis

The data were manually checked for completeness and entered into Statistical Package for the Social Sciences (SPSS) Version 25 software for analysis. Descriptive statistics, such as frequency distributions in relation different variables were determined. Binary logistic regression analyses were also performed to assess the association of different variables with intestinal parasite infection. Those variable with *p*-value >0.25 in the bivariate logistic regression were fitted to multivariable logistic regression. Statistical test results were declared significant when the 𝑃- value was <0.05 and rough and adjusted odds ratios with 95% confidence intervals (CIs) were reported.

## Results

### Socio-demographics of the study subjects

A total of 191 people living with HIV/AIDS were participated in the study. Among these, 113 (59.2%) were females, and 89 (46.6%) were between the ages of 35 to 50 years. Of the total study participants, 103 (53.9%) were urban residents and 133 (69.6%) were married. O the other hand 65 (34.0%) of the study participants had completed their primary school, and 44 (23.0%) were working as housewives (Table 1).

**Table 1:** Prevalence of intestinal parasites with socio-demographic characteristics of people living with HIV/AIDS (*N* = 191).

Characteristics	Total *N* (%)	Parasitic infections		COR (95% CI)	AOR (95% CI)	*p*-value
		Positive *N* (%)	Negative *N* (%)			
Age groups						
18–34	67 (35.1)	24 (35.8)	43 (64.2)	1.07 (0.45–2.52)	―	0.89
35–50	89 (46.6)	31 (34.8)	58 (65.2)	1.02 (0.45–2.33)	―	0.95
> 50	35 (18.3)	12 (34.3)	23 (65.7)	1		
Gender						
Male	78 (40.8)	31 (39.7)	47 (60.3)	1.41 (0.77–2.57)		
Female	113 (59.2)	36 (31.9)	77 (68.1)	1	―	0.26
Residence						
Urban	103 (53.9)	27 (26.2)	76 (73.8)	1		
Rural	88 (46.1)	40 (45.5)	48 (54.5)	2.35 (1.28–4.31)	2.34 (1.27–4.32)	0.01*
Marital status						
Married	133 (69.6)	41 (30.8)	92 (69.2)	1		
Single	58 (30.4)	26 (44.8)	32 (55.2)	1.82 (0.97–3.44)	―	0.06
Educational status						
Illiterate	14 (7.3)	5 (35.7)	9 (64.3)	1		
Read and write	41 (21.5)	15 (36.6)	26 (63.4)	1.44 (0.32–6.49)	―	0.63
Primary school	65 (34.0)	23 (35.4)	42 (64.6)	1.50 (0.45–5.03)	―	0.51
Secondary school	53 (27.7)	19 (35.8)	34 (64.2)	1.42 (0.45–4.50)	―	0.55
College and above	18 (9.4)	5 (27.8)	13 (72.2)	1.45 (0.45–4.70)	―	0.53
Occupation						
Gov’t employer	27 (14.1)	6 (22.2)	21 (77.8)	1		
Farmer	36 (18.8)	14 (38.9)	22 (61.1)	2.23 (0.71–6.90)	―	0.16
Housewife	44 (23.0)	16 (36.4)	28 (63.6)	2.00 (0.67–5.98)	―	0.22
Merchants	34 (17.8)	12 (35.3)	22 (64.7)	1.91 (0.61–6.02)	―	0.27
Student	9 (4.7)	4 (44.4)	5 (55.6)	2.80 (0.57–13.83)	―	0.21
Daily laborers	41 (21.5)	15 (36.6)	26 (63.4)	2.02 (0.67–6.11)	―	0.21

### Intestinal parasites in ART and ART naïve group

In this study, a total of 191 stool samples were tested for the detection of intestinal parasites (IPs). Of these, 143 (75.0%) were taken from people living with HIV/AIDS who were on ART, and the rest, 48 (25.0%), were taken from the ART naïve group. The overall prevalence of IPs was 67 (35.1%). The prevalence of IPs among people living with HIV/AIDS who were on ART was 45 (31.5%) and 22 (45.8%) among the ART naïve group. The distribution of intestinal parasites showed no statistically significant differences between the ART and ART naïve groups (X^2^ = 3.256; *P*-value >0.05). The prevalence of protozoa, helminths, and opportunistic parasites were 21/143 (14.7%), 22/143 (15.4%), and 2/143 (1.4%) among ART-started patients, and 11/48 (22.9%), 8/48 (16.7%), and 3/48 (6.25%) among ART naïve patients, respectively. *Entamoeba histolytica/dispar* was the most prevalent parasite detected from protozoan parasites in both ART and ART naïve patients, which was, 7.7 and 12.5%, respectively. At the same time, *Ascaris lumbricoides* was the most prevalent parasite detected from helminths in both ART and ART naïve patients, which was 6.3 and 6.25%, respectively. From opportunistic parasites, the prevalence of *Cryptosporidium* spp. among ART and ART naïve patients was 1.4 and 4.2%, respectively, however, *Isospora belli* (2.1%) was detected only in the ART naïve group (Table 2).

**Table 2:** Prevalence of intestinal parasites with ART status.

Parasites detected	ART status		X^2^ (*p*-value)
	On-ART *N* (%)	ART naïve group *N* (%)	
Protozoans			
*Entamoeba histolytica/dispar*	11 (7.7)	6 (12.5)	0.26
*Giardia lamblia*	10 (7.0)	5 (10.4)	0.65
Sub-total *N* (%)	21/143 (14.7)	11/48 (22.9)	0.27
Helminths			
*Ascaris lumbricoides*	9 (6.3)	3 (6.25)	0.99
*Trichuris trichiura*	4 (2.8)	2 (4.2)	0.44
*Hymenolepis nana*	2 (1.4)	0	0.41
*Schistosoma mansoni*	4 (2.8)	2 (4.2)	0.44
*Strongyloides stercolaris*	1 (0.7)	0 (0.0)	0.56
*Hookworm*	1 (0.7)	1 (2.1)	0.44
*Taenia* spp.	1 (0.7)	0 (0.0)	1.00
Sub-total *N* (%)	22/143 (15.4)	8/48 (16.7)	1.00
Opportunistic parasite			
*Cryptosporidium* spp.	2 (1.4)	2 (4.2)	0.25
*Isospora belli*	0 (0.0)	1 (2.1)	0.08
Sub-total *N* (%)	2/143 (1.4)	3/48 (6.25)	0.10
Double infections	3 (2.1)	2 (4.2)	―
Total *N* (%)	45 (31.5)	22 (45.8)	0.07

### Intestinal parasites regarding socio-demographics of the study participants

In this study, analysis with binary logistic regression showed that the place of residence of the study participants was identified as the only socio-demographic determinant of intestinal parasite infections among people living with HIV/AIDS. Intestinal parasites were detected in 40 (45.5%) of rural residences, which was 2.34 times (AOR = 2.34; 95% CI: 1.27–4.32; *p*-value = 0.01) greater than those of urban residences. The other socio-demographic characteristics, such as gender, age, marital status, and educational status, were not associated with intestinal parasite infection (Table 1).

### Intestinal parasites regarding CD4 level, viral load, and ART status

In this study, from clinical findings, having CD4 counts <200 cells/mm^3^ was the only factor significantly associated with intestinal parasitic infection. As shown in Table 3, people living with HIV/AIDS whose CD4 counts <200 cells/mm^3^ was 3.77 times (AOR = 3.77; 95% CI: 1.01–13.15; *p*-value = 0.04) more likely to get an infection with intestinal parasites than those with CD4 counts >500 cells/mm^3^ (Table 3).

**Table 3:** Association of intestinal parasite prevalence with CD4 count, viral load, and ART status in people living with HIV/AIDS.

Variables	Total *N* (%)	Intestinal parasites		COR (95% CI)	AOR (95% CI)	*p*-value
		Positives *N* (%)	Negative *N* (%)			
CD4 levels						
<200cell/mm^3^	15 (7.9)	8 (53.3)	7 (46.7)	3.28 (1.07–10.05)	3.77 (1.01–13.15)	0.04*
200–500 cell/mm^3^	87 (45.5)	36 (41.4)	51 (58.6)	2.03 (1.07–3.83)	1.89 (0.97–3.64)	0.06
>500cell/mm^3^	89 (46.6)	23 (25.8)	66 (74.2)	1	1	
ART status						
On ART	143 (74.9)	45 (31.5)	98 (68.5)	1	1	
ART naïve	48 (25.1)	22 (45.8)	26 (54.2)	1.83 (0.94–3.60)	―	0.07
Viral load						
< 150 Copies/ml	129 (67.5)	40 (23.7)	89 (76.3)	1	1	
≥150 Copies/ml	62 (32.5)	27 (42.9)	35 (57.1)	1.72 (0.92–3.21)	―	0.09

NB: *: statistical significance association.

### Intestinal parasites regarding hygienic practices of the study participants

In this study, among the studied variables related to the environmental and hygienic practices of study participants, consuming unwashed raw vegetables was significantly associated with intestinal parasitic infection. The odds of having an intestinal parasitic infection in people eating unwashed raw vegetables were 3.29 times (AOR = 3.29; 95% CI: 1.23–8.86; *p*-value = 0.02) higher than those not consuming unwashed raw vegetables (Table 4).

**Table 4:** Association of intestinal parasite prevalence with environmental conditions and hygienic practices among HIV-positive patients (*N* = 191).

Variables	Total *N* (%)	Parasitic infections		COR (95% CI)	AOR (95% CI)	*p*-value
		Positive *N* (%)	Negative *N* (%)			
Presence of toilet						
Yes	180 (94.2)	63 (35)	117 (65)	1		
No	11 (5.8)	4 (36.4)	7 (63.6)	1.06 (0.30–3.76)	―	0.93
Eating meals without hand washing						
Yes	9 (4.7)	4 (44.4)	5 (55.6)	1.51 (0.39–5.83)	―	0.55
No	182 (95.3)	63 (34.6)	119 (65.4)	1		
Source of water						
Tape water	171 (89.5)	59 (34.5)	112 (65.5)	1		
River/spring water	20 (10.5)	8 (40.0)	12 (60.0)	1.27 (0.49–3.27)	―	0.63
Frequent contact with animal feces						
Yes	55 (28.8)	20 (36.4)	35 (63.6)	1.08 (0.56–2.08)	―	0.81
No	136 (71.2)	47 (34.6)	89 (65.4)	1		
Frequent consumption of raw food						
Yes	181 (94.8)	66 (36.5)	115 (63.5)	5.16 (0.64–41.67)	―	0.12
No	10 (5.2)	1 (10.0)	9 (90.0)	1		
Frequent eating habit of unwashed vegetables						
Yes	38 (19.9)	20 (52.6)	18 (47.4)	2.51 (1.22–5.17)	3.29 (1.23–8.86)	0.02*
No	153 (80.1)	47 (30.7)	106 (69.3)	1	1	
Frequent contact with any water body						
Yes	48 (25.1)	14 (29.2)	34 (70.8)	0.70 (0.34–1.42)	―	0.32
No	143 (74.9)	53 (37.1)	90 (62.9)	1		
Frequently walking barefoot						
Yes	17 (8.9)	7 (41.2)	10 (58.8)	1.33 (0.48–3.67)	―	0.58
No	174 (91.1)	60 (34.5)	114 (65.5)	1		

NB: *: statistical significance association.

## Discussion

This hospital-based cross-sectional study was conducted to assess the prevalence of intestinal parasites among people living with HIV/AIDS in the southwest district of Ethiopia. The observed prevalence of intestinal parasites among HIV/AIDS-infected individuals was found to be 35.1%, indicating high rates of parasitic infections in this population. The high prevalence of intestinal parasites among HIV-infected individuals is also reported in various African regions, particularly sub-Saharan Africa. For instance, a study conducted in Nigeria reported 68.2% (22), Gabon 42.6% (23), Cameroon 57.5% (24), and Kenya 50.9% (25). Additionally, a similar study from Ethiopia reported rates of 73.3% in Nekemte (26) and 45.3% in Gondar (27), underscoring the widespread nature of these infections across the region. These trends suggest that intestinal parasitic infections continue to be a significant burden among HIV-infected populations, especially in areas where ART access is limited and sanitation practices are inadequate.

In contrast, lower prevalence rates have been reported in other areas. For instance, a study conducted Mozambique reported 26.4% (28), India 27.6% (29), and Colombia 29.2% (30). This difference might be attributed to the widespread availability of ART and improved public health measures, including better sanitation and hygiene practices, in these settings. The lower prevalence in such countries highlights the critical role that ART and robust healthcare systems play in reducing the burden of opportunistic infections.

Moreover, in this study, the prevalence of intestinal parasites in the ART and ART naïve groups was (31.5%) and (45.8%) respectively, which was in line with a study finding in Northern Ethiopia, in which a higher proportion of intestinal parasites was observed in the ART naïve groups (27, 31). This consistent trend across multiple studies underscores the protective effect of ART in mitigating the risk of parasitic infections by improving immune function. However, the persistent high prevalence even among ART-treated patients in our study suggests that other factors, such as environmental conditions, hygiene practices, and possibly suboptimal ART adherence or effectiveness, also play a critical role in the transmission and maintenance of these infections (32).

Although there were no statistically significant differences in overall parasite species detected between the ART and ART-naive groups, in the present study, *Entamoeba histolytica/dispar* and *Giardia lamblia* were the most prevalent parasites detected both in the ART and ART-naïve groups. However, the prevalence of *Cryptosporidium* spp. in ART and ART naïve groups was 1.4 and 4.2%, respectively. This finding is in line with other study findings in Ethiopia (18, 27, 31) and Colombia (30), in which a higher predominance of opportunistic intestinal parasites was reported. The higher proportion of opportunistic intestinal parasites in ART naïve groups indicates that ART may also contribute to the reduction of opportunistic intestinal parasite infections.

In this study, intestinal parasitic infection occurrence was significantly higher in patients with a CD_4_ count of less than 200 cells/mm^3^, which is in line with other study findings from different parts of Ethiopia (33–36). Another study in Ethiopia reported that a CD_4_ count of less than 500 cells/mm^3^ was significantly associated with opportunistic intestinal parasitic infections (37). The low level of CD4 count leads to weakened immune responses and increases their chances of getting infections that the body would normally fight off very easily. Immuno-deficient patients are more vulnerable to acquiring intestinal parasites and are unable to clear the infection once it is established (36, 38). Some studies reported that a baseline CD4 count of ≥500 cells/μl was significantly associated with viral load reductions or suppression (39–41).

In this study a significant association between the consumption of unwashed raw vegetables and a higher prevalence of intestinal parasites were identified. This association consuming of contaminated food with intestinal parasitic infections is a known associated factor as reported in several other studies (42, 43). For instance, a study conducted in Brazil showed that HIV-infected individuals who consumed raw vegetables were twice as likely to be infected with intestinal parasites compared to their counterpart (44). In areas where there is contamination open field with human excreta, the risk of contamination is particularly high. Public health interventions that emphasis on educating HIV-infected individuals about safe food handling practices, as well as improving the quality of water used for washing vegetables, are essential in reducing the burden of intestinal parasitic infections.

Furthermore, the study also identified that a significant association between living in rural area with a higher prevalence of intestinal parasites. This study finding is in agreement with other studies from low-resource areas, where rural populations often have limited access to clean water, sanitation, and healthcare services (45). For instance, a study in rural Uganda, reported a similar association, contributing to the higher prevalence of intestinal parasitic infections to the lack of infrastructure and health education in rural communities (46). The disparity in the prevalence of parasitic infections in different geographic areas underscores the need for targeted interventions that address the specific challenges faced by rural populations, such as improving access to healthcare, sanitation, and clean water.

In the present study, only saline and iodine wet mounts, formaldehyde-ether concentrations, and modified acid-fast staining were used to detect intestinal parasites. Thus, the added yield of intestinal parasites may be an underestimate as we have not used other methods like molecular techniques and immunofluorescent techniques, which are sensitive to parasites. Additionally, patients may have received deworming and/or been diagnosed with parasites and treated as well before.

## Conclusion

The study revealed a high prevalence of intestinal parasite infections among adults living with HIV/AIDS, with a higher infection rate observed in ART-naïve patients compared to those on ART. The findings indicate that lower CD4 counts, consumption of unwashed raw vegetables, and residing in rural areas are significant risk factors for intestinal parasitosis in this population. These results underscore the need for targeted interventions, including improving ART access, promoting better hygiene practices, especially regarding food safety, and focusing on vulnerable populations in rural areas. Regular screening for intestinal parasites in HIV-infected individuals, particularly those with low CD4 counts, is also recommended to reduce morbidity and improve patient outcomes.

## Data Availability

The original contributions presented in the study are included in the article/supplementary material, further inquiries can be directed to the corresponding author.
